# Supplementary material for: Diesel2p mesoscope with dual independent scan engines for flexible capture of dynamics in distributed neural circuitry
Source: Nat Commun. 2021 Nov 17;12:6639. doi: 10.1038/s41467-021-26736-4 (PMC8599518; doi:10.1038/s41467-021-26736-4)
Supplement: Supplementary file 1 — Supplementary Information [file 41467_2021_26736_MOESM1_ESM.pdf]

Diesel2p mesoscope with dual independent scan engines for flexible capture of dynamics in distributed neural circuitry

Che-Hang Yu[1]ǂ, Jeffrey N. Stirman[2]ǂ, Yiyi Yu[1], Riichiro Hira[1], Spencer L. Smith[1]*

[1]Department of Electrical and Computer Engineering, University of California, Santa Barbara, CA, USA

[2] LifeCanvas Technologies, Cambridge, MA, USA

ǂ These authors contributed equally

**Corresponding author: sls@ucsb.edu*

Supplementary Figure 1

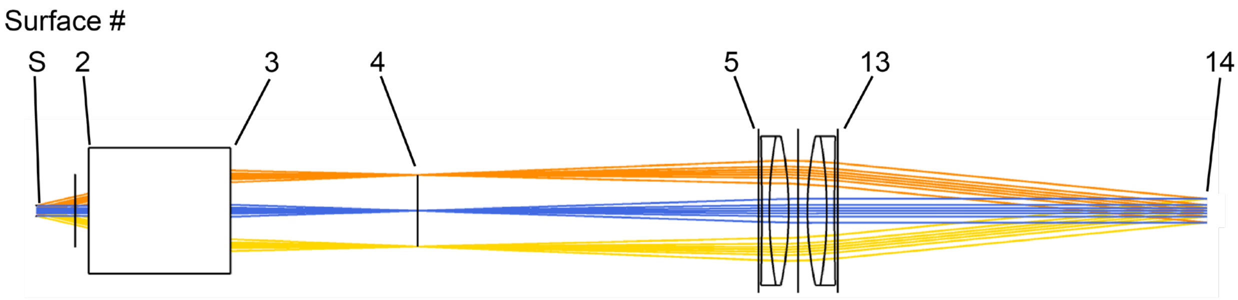

Surface #	Description	Radius (mm)	Thickness (mm)	Material	Semi-Diameter
Stop	X-RM	Inf	13.25		
1	Thread	Inf	4.7		12.5
2	LSM54-1050.ZBB		48.3		21.5
3	Working distance	Inf	63.912		21.5
4		Inf	116.288		12.228
5	Outer Housing	Inf	1.1		28.0
6	AC508-250-B-ML	-1235.9	2.6	N-SF6HT	25.4
7		146.140	6.6	N-BAF10	25.4
8		-121.22	3.2		25.4
9		Inf	3.2		28.0
10	AC508-250-B-ML	121.220	6.6	N-BAF10	25.4
11		-146.14	2.6	N-SF6HT	25.4
12		1235.9	1.1		25.4
13	Outer Housing	Inf	126.550		28.0
14	X-GM	Inf	next page		

Thorlabs   Assembly compensator

Supplementary Figure 1. Full prescription data for X-resonant mirror (X-RM) to X-galvo mirror (X-GM) relay
This optical relay was constructed using commercial off-the-shelf (COTS) components from Thorlabs. The red outlined axial separation are used as compensators in system assembly.

Supplementary Figure 2

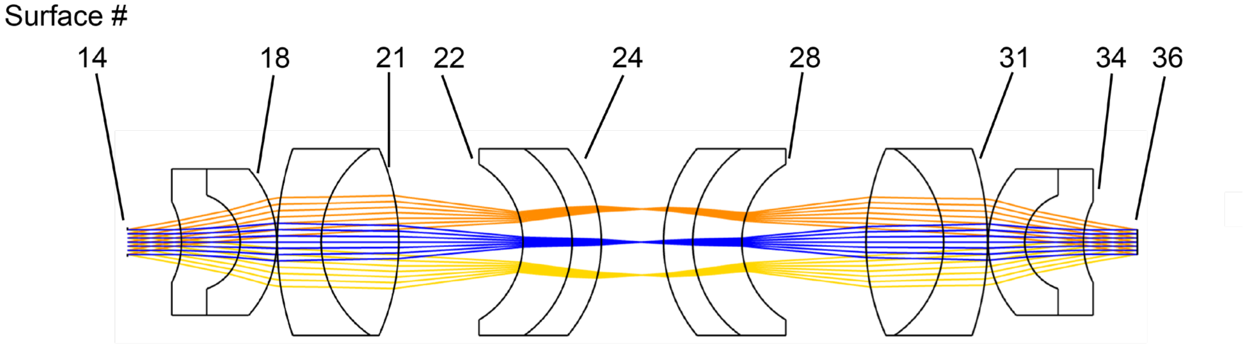

Surface #	Description	Radius (mm)	Thickness (mm)	Material	Semi-Diameter
14	X-GM	Inf	15.458		
15		Inf	1.937		10.375
16		-28.758	18.905	S-PHM52	13.0
17		-15.850	11.727	N-SF57	15.0
18		-35.283	0.25		23.5
19		92.835	14.141	N-SF57	30.0
20		36.302	25.009	S-LAH53	30.0
21		-73.561	39.995		30.0
22		-29.129	15.824	N-SF57	25.0
23		-36.881	9.427	N-BK7	30.0
24		-46.980	10.0		30.0
25		Inf	10.0		10.805
26		46.980	9.427	N-BK7	30.0
27		36.881	15.824	N-SF57	30.0
28		29.129	39.995		25.0
29		73.561	25.009	S-LAH53	30.0
30		-36.302	14.141	N-SF57	30.0
31		-92.835	0.25		30.0
32		35.283	11.727	N-SF57	23.5
33		15.850	18.905	S-PHM52	15.0
34		28.758	1.937		13.0
35		Inf	15.458		10.375
36	Y-GM	Inf	next page		

Assembly compensator

Supplementary Figure 2. Full prescription data for X-galvo mirror (X-GM) to Y-galvo mirror (Y-GM) relay
The 6 elements from the surface 16 to 24 form a scan-lens subassembly with an effective focal length of 61 mm. The surfaces of 26-34 is the exact reverse of 16-24, and also have the same effective focal length of 61 mm. The red outlined axial separation are used as compensators in system assembly.

Supplementary Figure 3

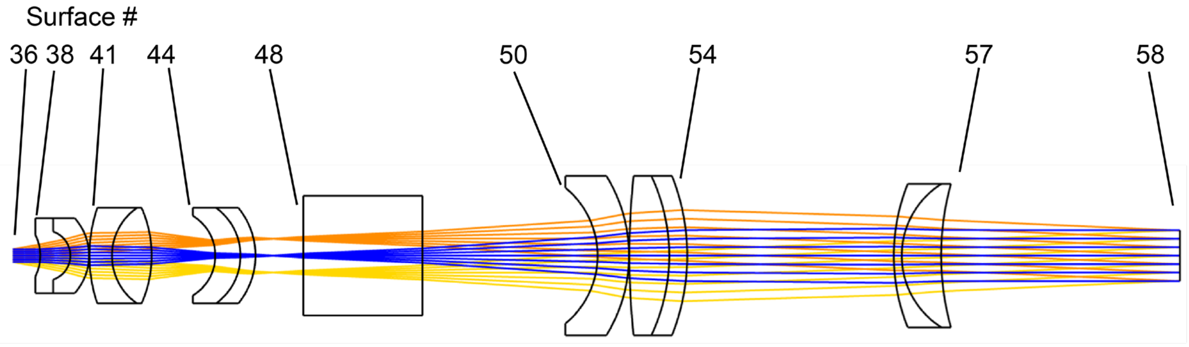

Surface #	Description	Radius (mm)	Thickness (mm)	Material	Semi-Diameter
36	Y-GM	Inf	15.458		
37		Inf	1.937		10.375
38		-28.758	18.905	S-PHM52	13.0
39		-15.850	11.727	N-SF57	15.0
40		-35.283	0.25		23.5
41		92.835	14.141	N-SF57	30.0
42		36.302	25.009	S-LAH53	30.0
43		-73.561	39.995		30.0
44		-29.129	15.824	N-SF57	25.0
45		-36.881	9.427	N-BK7	30.0
46		-46.980	10.0		30.0
47		Inf	20.0		10.652
48	PBS	Inf	75.0	BK7	37.5
49		Inf	110.032		37.5
50		-54.856	19.753	N-BK7	42.5
51		-94.92	0.25		50.0
52		471.246	25.0	S-PHM52	50.0
53		-117.056	11.366	LF5	50.0
54		-134.483	129.824		50.0
55		119.545	4.991	LF5	45.0
56		53.014	24.765	S-FPL51	45.0
57		167.417	150.0		45.0
58	Obj	Inf	next page		

Assembly compensator

Supplementary Figure 3. Full prescription data for system from the Y-galvo mirror (Y-GM) to the objective
The 6 elements from the surface 38 to 46 also form a scan-lens subassembly with an effective focal length of 61 mm. The 6 elements from the surface 50 to 57 form the tube-lens sub-assembly with an effective focal length of 243 mm. The polarizing beam splitter (PBS) was offset from the focal point of the relay to minimize photo-damage to the optical cement. The terminal lens in this system has a 90 mm diameter in order to minimize vignetting at the extreme scan angles. The red outlined axial separation are used as compensators in system assembly.

Supplementary Figure 4

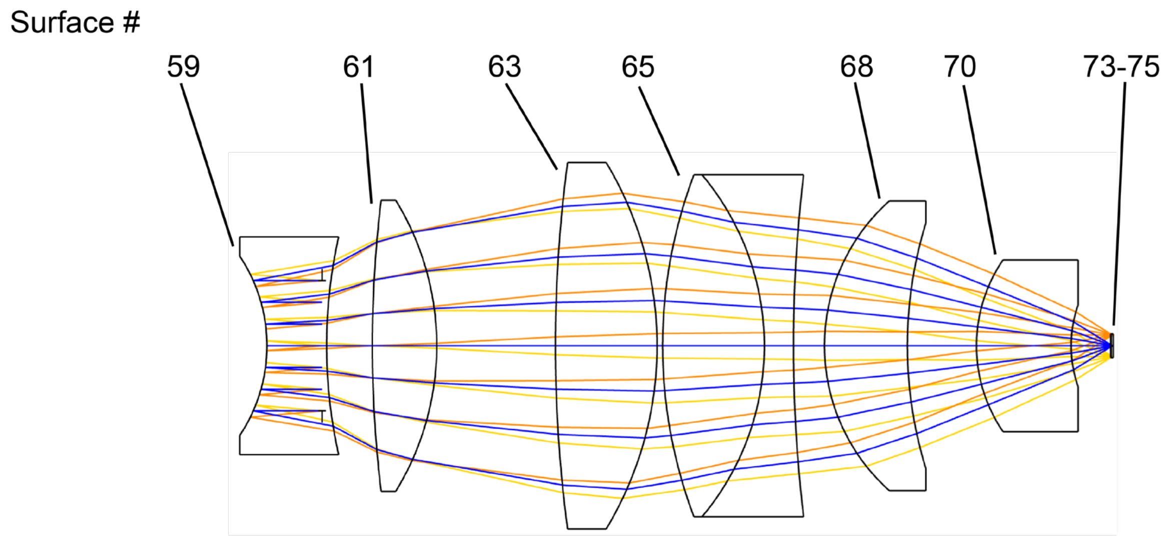

Surface #	Description	Radius (mm)	Thickness (mm)	Material	Semi-Diameter
58	Obj	Inf	-13.53		
59		-39.472	14.678	S-TIH6	22.0
60		121.92	11.247		26.75
61		299.796	15.845	N-SK2	35.75
62		-68.377	29.202		35.75
63		340.055	25.0	N-SK2	45.0
64		-87.103	1.379		45.0
65		118.661	25.0	S-PHM52	42.0
66		-65.237	7.171	S-TIH53	42.0
67		352.311	7.637		42.0
68		47.688	20.378	S-PHM52	35.6
69		102.060	17.002		30.2
70		37.297	23.286	S-TIH53	21.1
71		30.348	1.695		10.0
72		Inf	8.0		7.962
73	Coverslip	Inf	0.15	BK7	2.906
74	Tissue	Inf	0.5	SEAWATER	2.849
75	Image plane	Inf			

Assembly compensator

Supplementary Figure 4. Full prescription data for an infinity-corrected, f = 30 mm, NA = 0.54 objective
The objective has ~8 mm of working distance and 30-mm focal length. The axial separation on surface 67 (red outline) is used as a compensator in the objective assembly. Focus compensation occurs at surface 72.

Supplementary Figure 5

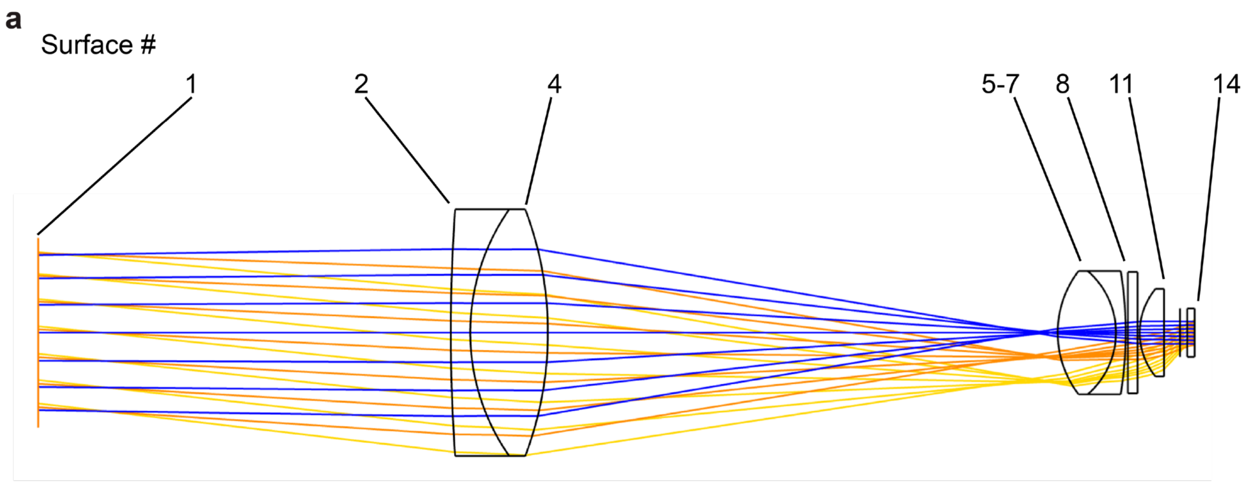

Surface #	Description	Radius (mm)	Thickness (mm)	Material	Semi-Diameter
1	Obj back aperture	Inf	85		19.503
2	AC508-100-A-ML	363.10	4	SF10	25.4
3		44.170	16	N-BAF10	25.4
4		-71.12	105		25.4
5	AC254-030-A-ML	20.89	12	N-BAF10	12.7
6		-16.730	2	N-SF6HT	12.7
7		-79.8	0.449		12.7
8	FESH0700	Inf	2	F-SILICA	12.5
9		Inf	0.449		12.5
10	48425	14.13	5	N-SF11	9
11		Inf	3.3		9
12	PMT housing	Inf	1.5		5
13	Window	Inf	1.5	BK7	5
14	Cathode	Inf			2.5

Thorlabs | Edmund | 5-mm diameter sensor

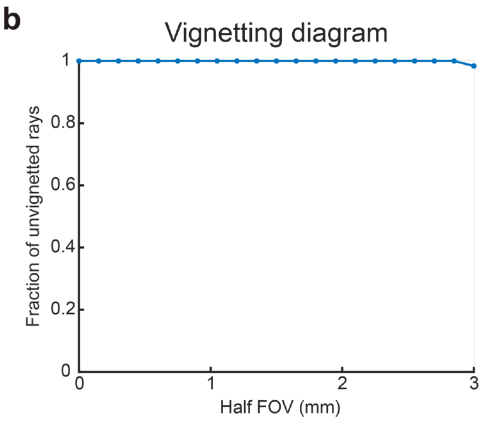

Supplementary Figure 5. Full prescription data and vignetting diagram for the collection relay

(a) This optical relay was constructed using COTS components from Thorlabs and Edmund. (b) The collection efficiency is nearly 100% up to a 3-mm half FOV (6-mm full FOV) with a sensor diameter of 5 mm.

Supplementary Figure 6

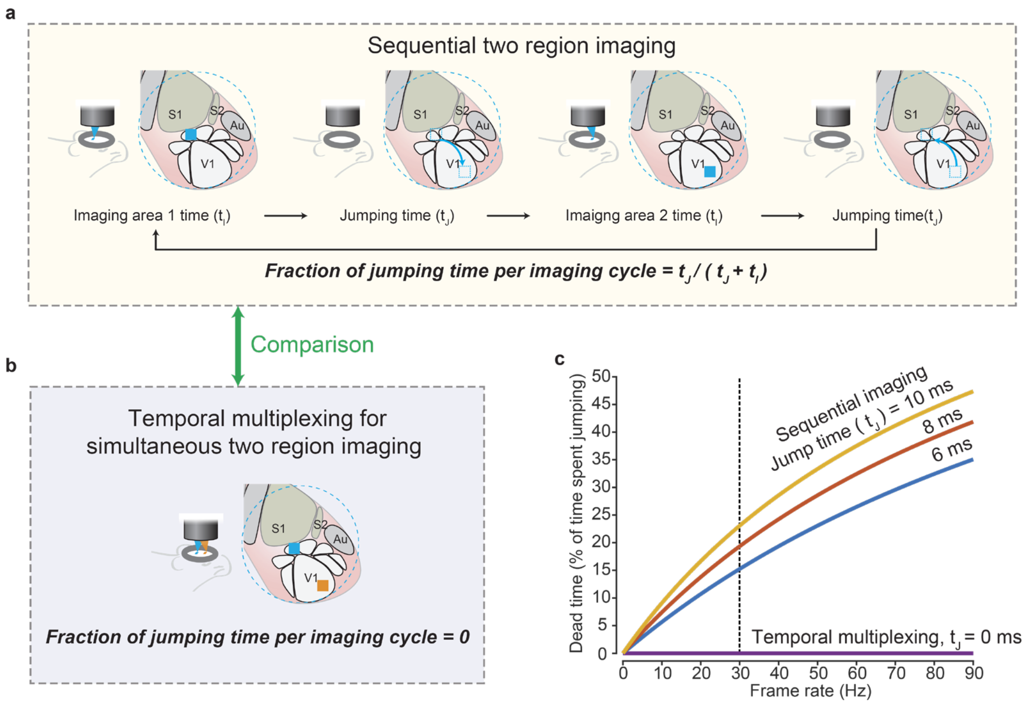

Supplementary Figure 6. Zero jumping time for simultaneous two-region imaging

(a) The cartoon shows the pipeline of the sequential imaging approach. Between two imaging acquisitions, a period of time is taken for the scanners to jump from a position to the next position. During an imaging cycle, a fraction of time is spent on the jumping.

(b) Zero fraction of time is spent on the jumping for temporal multiplexing imaging approach.

(c) A chart shows the fraction of jumping time versus the frame rate for the sequential imaging and the temporal multiplexed imaging. The higher the frame rate is, the larger fraction of the jumping time is for sequential imaging. The vertical dashed line indicated the 30-Hz frame rate, frequently referred to as real-time frame rate.

Supplementary Figure 7

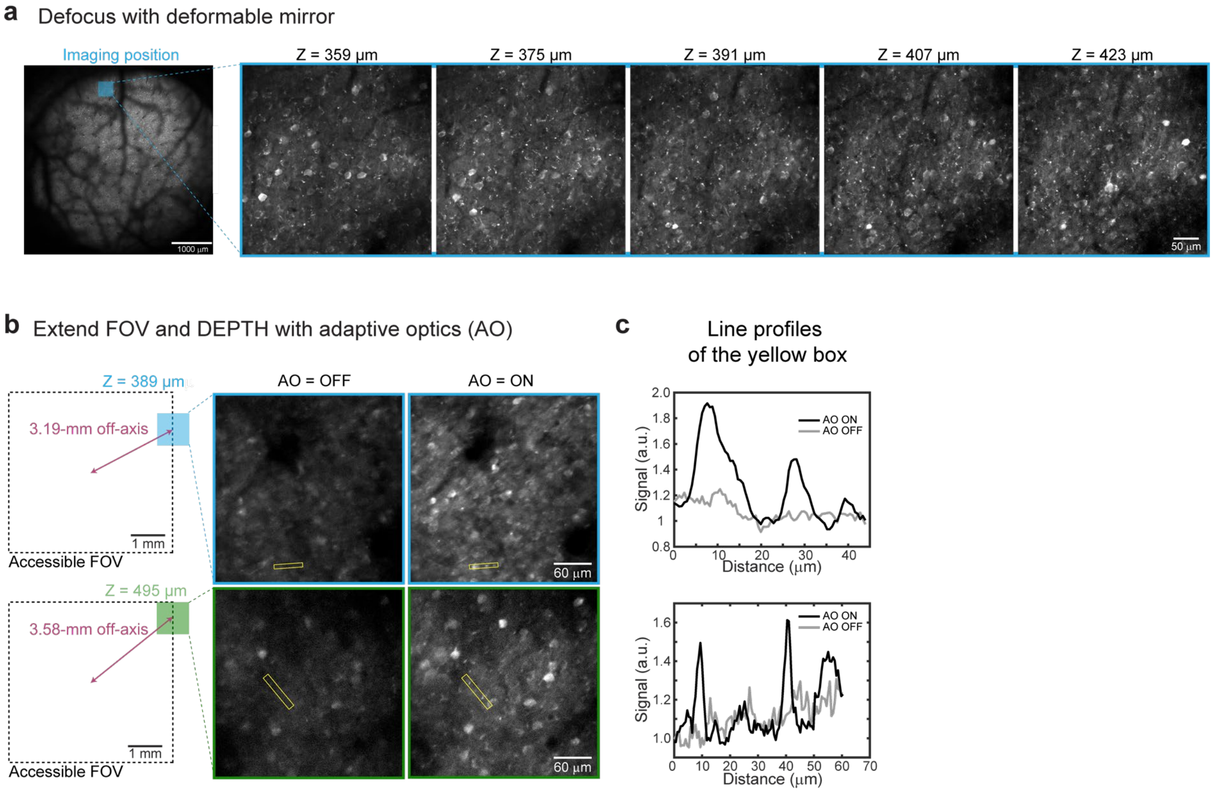

Supplementary Figure 7. Deformable mirrors extend the functionality and FOV

(a) Neuronal activity imaged at different depths by changing AO curvature. The lateral imaging position (blue) is shown on the full FOV (left). Images at each z plane is shown (right).

(b) Images measured at off-center positions >3 mm in the depths of 389 µm (blue) and 495 µm (green) without and with the AO correction. The image contrasts are set the same.

(c) Signal profiles in the yellow boxes in (b). Profiles are normalized to the noise level.

Supplementary Figure 8

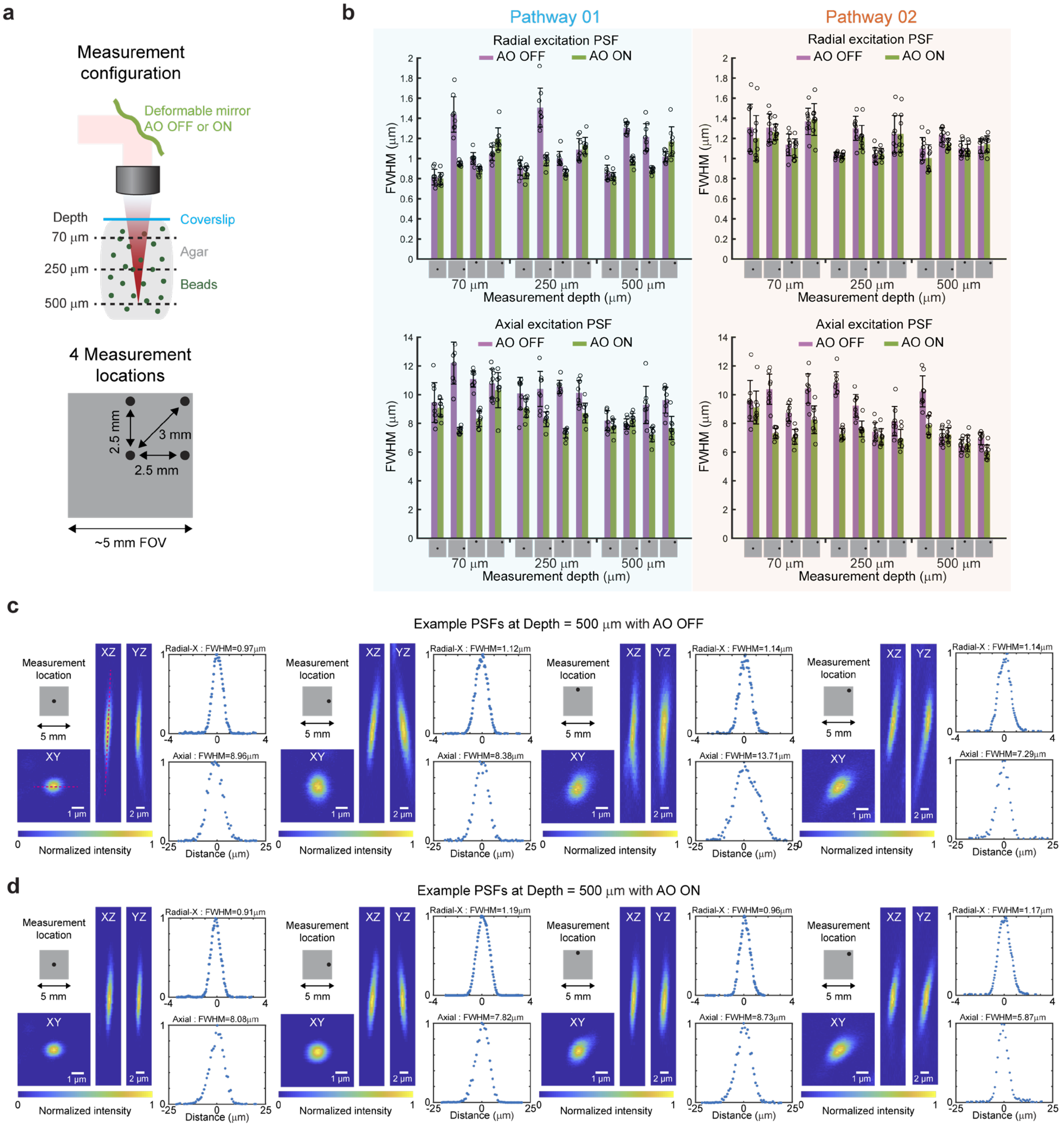

Supplementary Figure 8. Complete point spread function characterization of Diesel2p
(a) 0.2 µm fluorescent beads were embedded in 0.75% agarose gel. 40 µm z-stacks were acquired, each centered at one of three depths (70 µm, 250 µm, 500 µm). At each depth, beads at four lateral locations were measured: on axis, 2.5-mm off-axis near the two edges, and 3-mm off-axis diagonally near the corner of the FOV. At each position, measurements were done with the AO flattened (OFF) or deformed (ON).
(b) A complete summary of the excitation PSF measurements at positions indicated in (a) for both of the temporally multiplexed beam pathways with AO OFF and ON. FWHM of the Gaussian fits for measurements from the fluorescence beads radially and axially are calculated and plotted. Eight beads (n=8) were measured at most locations (the one exception is on axis at the depth of 500 µm, where seven (n=7) beads were measured). Data are presented as mean values ± S.D. The radial PSF is the average of both the radial FWHMs in the X and Y directions.

(c) Radial and axial excitation PSF volume measurements were made at the indicated locations and the depth of 500 µm where the example images are shown from the XY, XZ, and YZ cross-sections, respectively. The intensity profiles of the beads (red lines) in the X direction on the XY plane and in the Z direction are plotted, which are fitted to a Gaussian curve to extract the radial-X and axial full-width-half-maximum (FWHM) of the PSF. This row shows the measurements as the AO was set flat. (d) This row shows the same z-stack measurement from exact the same bead in (c) after the AO was applied and configured to maximize the fluorescence signal from that bead.

Supplementary Figure 9

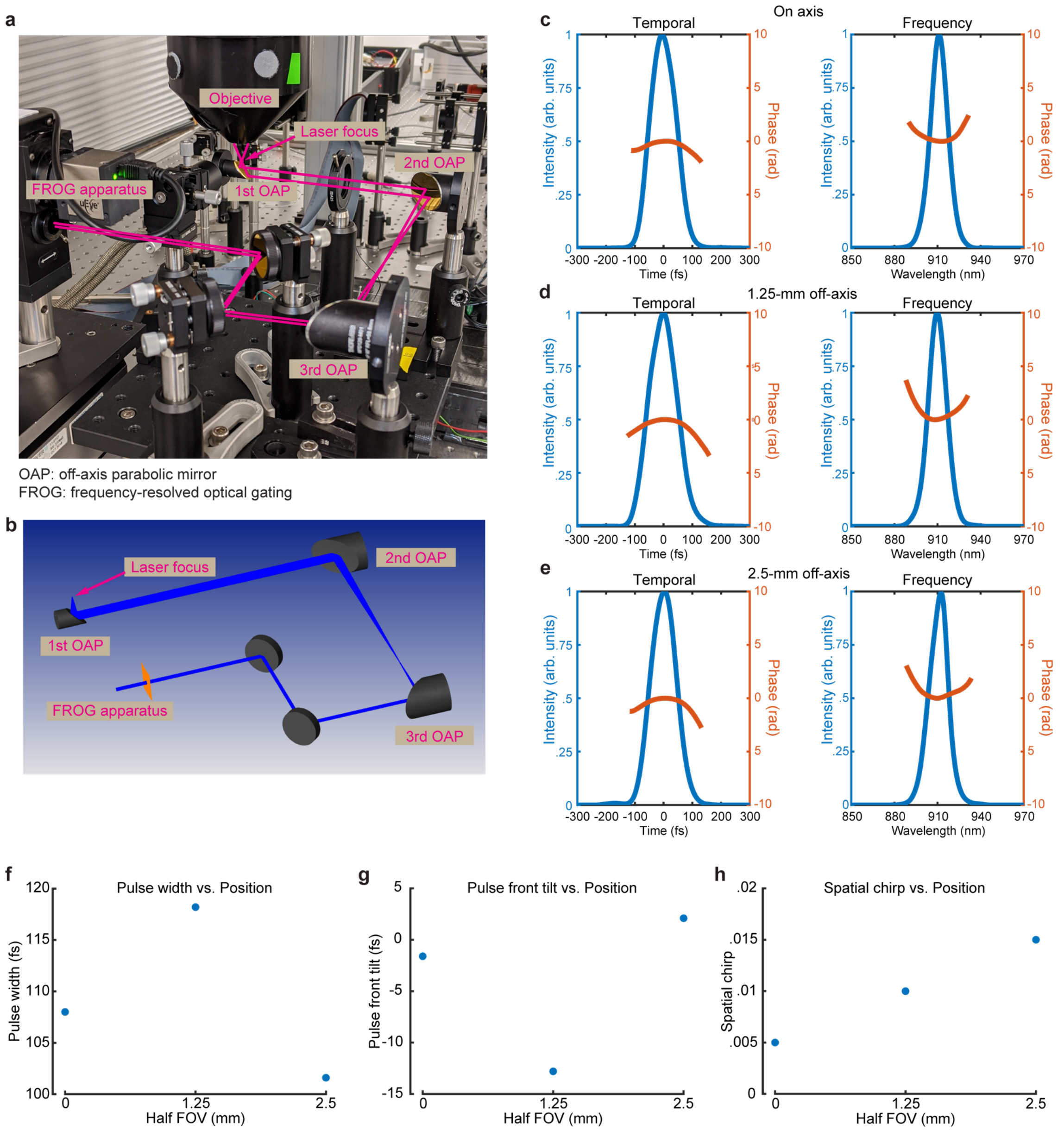

Supplementary Figure 9. Pulse characterization at the imaging plane
(a) A custom reflective relay redirected light from the focal plane to a pulse metrology device (FROG). (b) Optical model diagram of the beam path. (c-h) The temporal and the spatial measurement of pulses (c) at the center, (d) 1.25-mm off axis, and (e) 2.5-mm off-axis of the FOV. Overall, (f) the pulse width, (g) pulse front tilt, and (h) spatial chirp varied little over the full FOV (mean ± standard error from 5 measurements for each data point).

Supplementary Figure 10

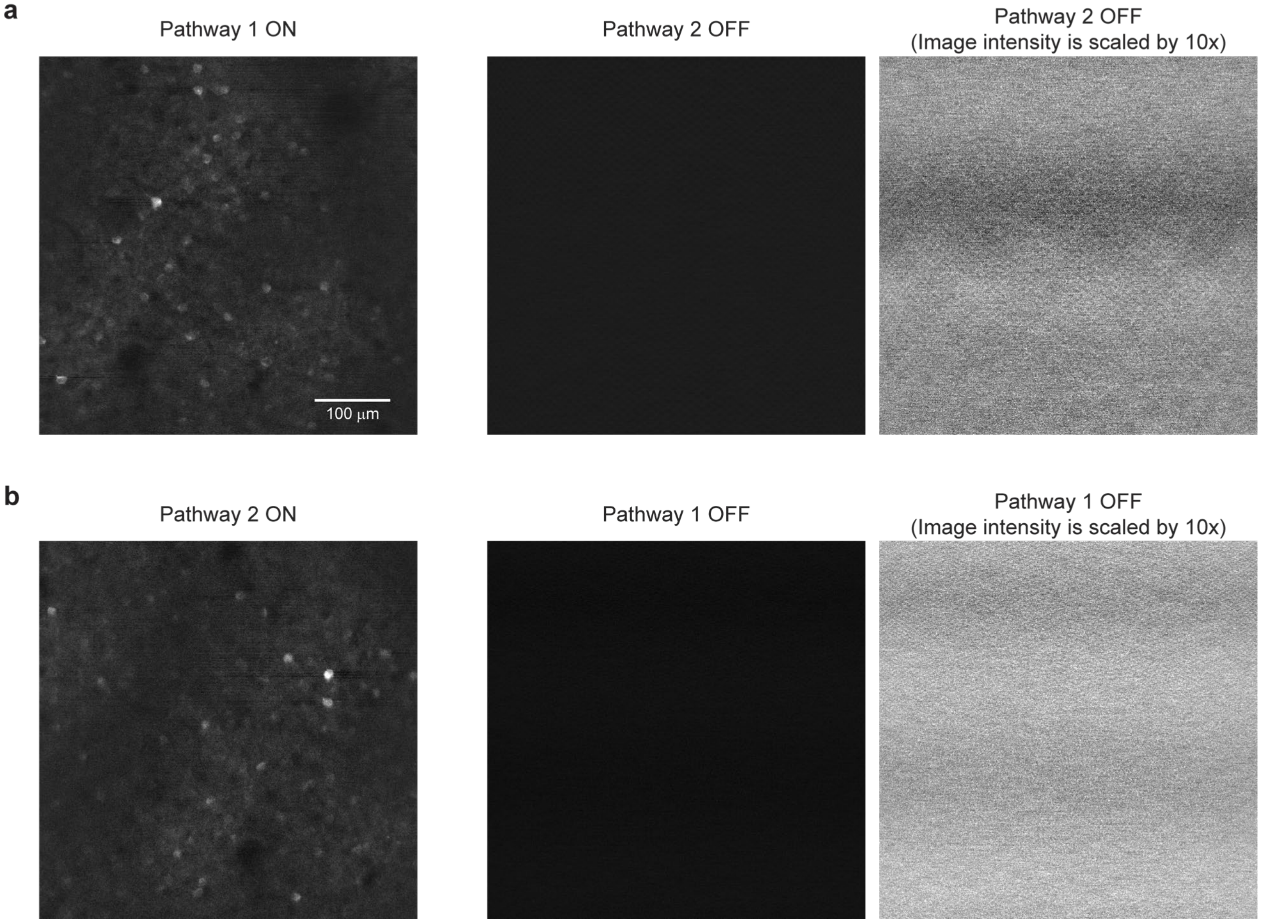

Supplementary Figure 10. Crosstalk is minimal between the two pathways
Crosstalk between the temporally multiplexed pathways was measured *in vivo* in a mouse expressing GCaMP6s. Neurons were (a) imaged with pathway 1 while blocking excitation in pathway 2, and (b) vice versa. The image intensity in the blocked pathway is scaled up 10-fold (the rightmost image in panels a and b). The result shows that the crosstalk is minimal, and there is no structured image bled through into the blocked channel. Since each independent pathway is run with a separate resonant scanner, which operate at slightly different frequencies, the structure of any residual bleedthough is desynchronized and thus there is no preserved structure to the bleedthrough. This result is in contrast to the temporally multiplexed pathways in a single-scan-engine system where crosstalk can result in structured bleedthrough between the imaging pathways.

Supplementary Figure 11

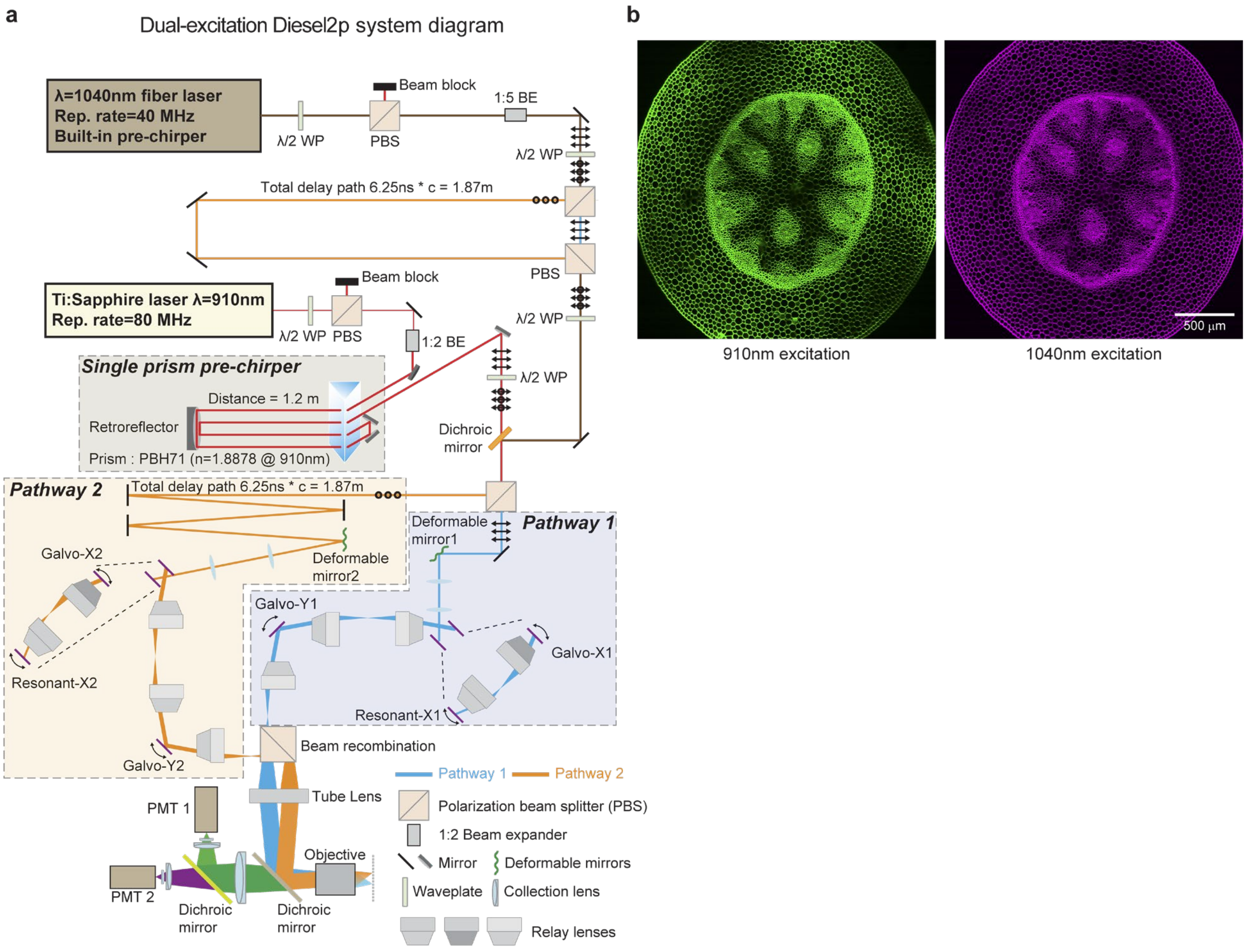

Supplementary Figure 11. Dual-excitation large FOV system and imaging
(a) A 1040-nm pulsed laser with a 40 MHz repetition rate is introduced into the system and merged with the 910-nm laser using a dichroic mirror. Before being merged with the 910-nm laser beam, the 1040-nm laser was temporally multiplexed by being split into two beamlets, and one beamlet was delayed from the other by ~ 12.5 ns (1/40MHz/2, using two 6.25 ns delay stages). After merged with the dichroic mirror, the two lasers were guided through the same optics downstream in the system, so that each pathway can use the two lasers simultaneously. Two photomultiplier tubes were used to collect the spectrally-separated emission photons excited by the 910-nm and 1040-nm lasers, individually. The signal collected by each photomultiplier tube is demultiplexed based on the laser arrival time, respectively, achieving a dual-band, dual-beamlet, dual-scan engine excitation and dual-channel collection system. In summary, there are four separate ultrafast pulse trains (two wavelengths, each with 2 beamlets) directed across the preparation using two independent scan engines (one for P-polarization beamlets, and one for S-polarization beamlets). With this configuration, both the 1040-nm laser and the 910-nm laser have simultaneous access to the full FOV of the two independent scan engines using temporally multiplexed foci. BE: Beam expander; WP: waveplate. (b) Two-photon fluorescence images of a fixed convallaria acquired with two excitation wavelengths, 910 nm and 1040 nm, respectively. The image size is 2500 x 2500 µm, acquired with a single raster scan without stitching. The same emission filter, a 700 nm short-pass filter, is used for the two images.

Supplementary Figure 12

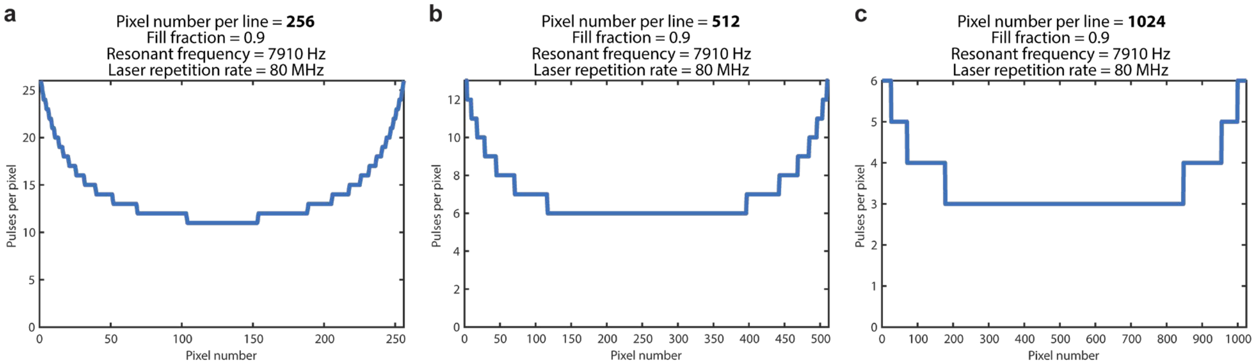

Supplementary Figure 12. Plots of laser pulses per pixel with the resonant scanner
The Diesel2p system is equipped with a 7910-Hz resonant scanner and a 910-nm pulse laser with a ~80-MHz repetition rate. The default fill fraction is 0.9 in our system. The pulses per pixel are plots, when the pixel number per line is (a) 256, (b) 512, and (c)1024. These three conditions are the major imaging parameters used in this paper.
