# Supplementary material for: Diesel2p mesoscope with dual independent scan engines for flexible capture of dynamics in distributed neural circuitry
Source: Nat Commun. 2021 Nov 17;12:6639. doi: 10.1038/s41467-021-26736-4 (PMC8599518; doi:10.1038/s41467-021-26736-4)
Supplement: Supplementary file 3 — Description of Additional Supplementary Files [file 41467_2021_26736_MOESM3_ESM.docx]

**Description of Additional Supplementary Files:**

**Supplementary Video 1:**

Rapid defocusing using AO. FOV: 375 x 375 µm, acquisition rate: 30 frames/s.

**Supplementary Video 2:**

Imaging enhancement using AO. FOV: 659 x 535 µm. acquisition rate: 30 frames/s.

**Supplementary Video 3:**

Simultaneous dual region large FOV (1.5 x 5.0 mm each) imaging at high pixel count (1024 x 4096 pixels each). Acquisition rate (3.85 frames/s). Depth: 371 µm.

**Supplementary Video 4:**

Simultaneous dual region imaging with flexible imaging configurations. Two regions are 4.36 mm apart. Pathway 1: FOV = 1500 x 1500 µm, acquisition rate = 15 frames/s, pixel count = 1024 x 1024 pixels, depth = 371 µm. Pathway 2: FOV = 256 x 256 µm, acquisition rate = 58 frames/s, pixel count = 256 x 256 pixels, depth = 371 µm.

**Supplementary Video 5:**

Simultaneous dual region imaging with part of regions overlapped. Pathway 1: FOV = 250 x 250 µm, acquisition rate = 60 frames/s, pixel count = 256 x 256 pixels, depth = 262 µm. Pathway 2: FOV = 750 x 750 µm, acquisition rate = 15 frames/s, pixel count = 1024 x 1024 pixels, depth = 262 µm.

**Supplementary Video 6:**

Quad region imaging with independent parameters and imaging planes. Pathway 1, Region 1 and Region 2: FOV = 1500 x 1500 µm, acquisition rate = 7.6 frames/s, pixel count = 1024 x 1024 pixels, depth = 361 µm. Pathway 2, Region 3 and Region 4: FOV = 250 x 250 µm, acquisition rate = 30 frames/s, pixel count = 256 x 256 pixels, depth = 371 µm.

**Supplementary Video 7:**

Random access imaging scan. Each cell body ROI is 21.5 x 21.5 µm, with 16 x 16 pixels. The set of ROIs is acquired 41 times each second, depth = 359 µm.
