# Supplementary material for: Diesel2p mesoscope with dual independent scan engines for flexible capture of dynamics in distributed neural circuitry
Source: Nat Commun. 2021 Nov 17;12:6639. doi: 10.1038/s41467-021-26736-4 (PMC8599518; doi:10.1038/s41467-021-26736-4)
Supplement: Supplementary file 11 — Reporting Summary [file 41467_2021_26736_MOESM11_ESM.pdf]

nature portfolio

Corresponding author(s): Spencer Smith

Last updated by author(s): Aug 17, 2021

Reporting Summary

Nature Portfolio wishes to improve the reproducibility of the work that we publish. This form provides structure for consistency and transparency in reporting. For further information on Nature Portfolio policies, see our Editorial Policies and the Editorial Policy Checklist.

Statistics

For all statistical analyses, confirm that the following items are present in the figure legend, table legend, main text, or Methods section.

n/a	Confirmed	
☐	☒	The exact sample size (*n*) for each experimental group/condition, given as a discrete number and unit of measurement
☐	☒	A statement on whether measurements were taken from distinct samples or whether the same sample was measured repeatedly
☒	☐	The statistical test(s) used AND whether they are one- or two-sided<br>*Only common tests should be described solely by name; describe more complex techniques in the Methods section.*
☒	☐	A description of all covariates tested
☒	☐	A description of any assumptions or corrections, such as tests of normality and adjustment for multiple comparisons
☐	☒	A full description of the statistical parameters including central tendency (e.g. means) or other basic estimates (e.g. regression coefficient) AND variation (e.g. standard deviation) or associated estimates of uncertainty (e.g. confidence intervals)
☒	☐	For null hypothesis testing, the test statistic (e.g. F, t, r) with confidence intervals, effect sizes, degrees of freedom and P value noted<br>*Give P values as exact values whenever suitable.*
☐	☒	For Bayesian analysis, information on the choice of priors and Markov chain Monte Carlo settings
☒	☐	For hierarchical and complex designs, identification of the appropriate level for tests and full reporting of outcomes
☐	☒	Estimates of effect sizes (e.g. Cohen's d, Pearson's r), indicating how they were calculated

Our web collection on statistics for biologists contains articles on many of the points above.

Software and code

Policy information about availability of computer code

Data collection	ScanImage 2019b (https://vidriotechnologies.com/scanimage/) was used for data collection.
Data analysis	All data analysis was carried out using MATLAB 2019. Calcium imaging data set were processed using Suite2p (suite2p.readthedocs.io) and spike inference was carried out using a Markov chain Monte Carlo methods (Pnevmatikakis, E. A. et al., 2013 Asilomar Conference on Signals, Systems and Computers). Any further information needed for replication can be provided upon request.

For manuscripts utilizing custom algorithms or software that are central to the research but not yet described in published literature, software must be made available to editors and reviewers. We strongly encourage code deposition in a community repository (e.g. GitHub). See the Nature Portfolio guidelines for submitting code & software for further information.

Data

Policy information about availability of data

All manuscripts must include a data availability statement. This statement should provide the following information, where applicable:

- Accession codes, unique identifiers, or web links for publicly available datasets
- A description of any restrictions on data availability
- For clinical datasets or third party data, please ensure that the statement adheres to our policy

The data set from this report are openly available in “figshare” at “http://doi.org/10.6084/m9.figshare.15163914”.

Field-specific reporting

Please select the one below that is the best fit for your research. If you are not sure, read the appropriate sections before making your selection.

☒ Life sciences ☐ Behavioural & social sciences ☐ Ecological, evolutionary & environmental sciences

For a reference copy of the document with all sections, see nature.com/documents/nr-reporting-summary-flat.pdf

Life sciences study design

All studies must disclose on these points even when the disclosure is negative.

Sample size	The purpose of this study is to demonstrate the functionality of a new microscopy technique, rather than drawing biological conclusions. One GCaMP6s mouse and one Thy-1 GFP mouse were used, and sufficient to show the microscope's capability.
Data exclusions	No data were excluded.
Replication	To determine resolution, 7-8 beads were imaged at each location in the field of view (Fig. 1f). As this study is to demonstrate the functionality of a microscopy technique, rather than drawing biological conclusions, replicate experiments were not performed.
Randomization	Animals in the colony were randomly chosen for this study. As this study is to demonstrate the functionality of a microscopy technique, rather than drawing biological conclusions, randomization does not apply to this study.
Blinding	No blinding was used for this study, as blinding is not required for this research.

Reporting for specific materials, systems and methods

We require information from authors about some types of materials, experimental systems and methods used in many studies. Here, indicate whether each material, system or method listed is relevant to your study. If you are not sure if a list item applies to your research, read the appropriate section before selecting a response.

Materials & experimental systems

n/a	Involved in the study	
☒	☐	Antibodies
☒	☐	Eukaryotic cell lines
☒	☐	Palaeontology and archaeology
☐	☒	Animals and other organisms
☒	☐	Human research participants
☒	☐	Clinical data
☒	☐	Dual use research of concern

Methods

n/a	Involved in the study	
☒	☐	ChIP-seq
☒	☐	Flow cytometry
☒	☐	MRI-based neuroimaging

Animals and other organisms

Policy information about studies involving animals; ARRIVE guidelines recommended for reporting animal research

Laboratory animals	We used GCaMP6s and Thy1-GFP Line O (Jackson Labs stock #007919) transgenic mice in this study. GCaMP6s transgenic mice were generated by triple crossing of TITL-GCaMP6s mice, Emxl-Cre mice (Jackson Labs stock #005628) and ROSA:LNL:tTA mice (Jackson Labs stock #011008). TITL-GCaMP6s mice were kindly provided by the Allen Institute. The GCaMP6s mouse is female at the age of ~350 days. The Thy-1 GFP mouse is male at the age of ~250 days. The mice were housed in 12 h dark/light reverse cycle room. Temperature set-point 74-76F; low-temperature alarm = 70F; high-temperature alarm = 78F. Relative Humidity ~45% (range 30-70%).
Wild animals	No wild animals were used in this study.
Field-collected samples	No field-collected samples were used in this study.
Ethics oversight	All procedures involving living animals were carried out in accordance with the guidelines and regulations of the US Department of Health and Human Services and approved by the Institutional Animal Care and Use Committee at University of California, Santa Barbara.

Note that full information on the approval of the study protocol must also be provided in the manuscript.
